# Small one-dimensional Euclidean preference profiles

**DOI:** 10.1007/s00355-020-01301-y

**Published:** 2021-02-10

**Authors:** Jiehua Chen, Sven Grottke

**Affiliations:** 1 TU Wien, Vienna, Austria; 2 TU Berlin, Berlin, Germany

## Abstract

We characterize one-dimensional Euclidean preference profiles with a small number of alternatives and voters. We show that every single-peaked preference profile with *two* voters is one-dimensional Euclidean, and that every preference profile with up to five alternatives is one-dimensional Euclidean if and only if it is both single-peaked and single-crossing. By the work of Chen et al.  (Social Choice and Welfare 48(2):409–432, 2017), we thus obtain that the smallest single-peaked and single-crossing preference profiles that are *not* one-dimensional Euclidean consist of three voters and six alternatives.

## Introduction

The *one-dimensional Euclidean* preference domain (also known as the unidimensional unfolding domain) is a spatial model of structured preferences which originates from economics (Hotelling 1929; Downs 1957), political sciences (Stokes 1963; Brams et al. 2002; Bogomolnaia and Laslier 2007), and psychology (Coombs 1964; Borg et al. 2018). In this domain, the alternatives and the voters are points in a one-dimensional Euclidean space, i.e., on the real line, such that the preference of each voter towards an alternative decreases as the Euclidean distance between them increases.

One-dimensional Euclidean preferences are necessarily single-peaked (Black 1948) and single-crossing (Roberts 1977) as proven by Coombs (1964), Doignon and Falmagne (1994), and Chen et al. (2017). The reverse, however, does not hold. In his work, Coombs (1964) provided a sample preference profile with 16 voters and 6 alternatives that is single-peaked and single-crossing, but *not* one-dimensional Euclidean. This counterexample seems quite large for real world scenarios. For instance, in rank aggregation or winner determination elections, one often either has few alternatives to begin with, or may consolidate by first making a shortlist of only a few alternatives out of many, which will be considered for the final decision. There are also settings where only a few voters are involved, as for instance in a hiring committee or when planning holidays for a family. Hence, a natural question arising in the context of one-dimensional Euclidean preferences is whether for profiles with less than 16 voters or less than 6 alternatives, being single-peaked and single-crossing is sufficient for being one-dimensional Euclidean. In other words, we are interested in the following question:

*What are the tight upper bounds on the number of alternatives or voters such that profiles within these bounds are one-dimensional Euclidean as long as they are single-peaked and single-crossing?*

We note that all three restricted preference domains (single-peakedness, single-crossingness, and one-dimensional Euclideanness) can be detected in polynomial time (Doignon and Falmagne 1994). While both, single-peakedness and single-crossingness, admit direct polynomial-time detection algorithms (Doignon and Falmagne 1994; Escoffier et al. 2008; Elkind et al. 2012; Bredereck et al. 2013), detecting one-dimensional Euclideanness is done via a non-trivial but polynomial-time reduction to linear programming (Doignon and Falmagne 1994; Knoblauch 2010; Elkind and Faliszewski 2014). Moreover, while both, single-peakedness and single-crossingness, can be characterized by a small finite set of forbidden subprofiles (Ballester and Haeringer 2011; Bredereck et al. 2013), one-dimensional Euclideanness does not have this finite characterization since Chen et al. (2017) showed that there are *infinitely* many single-peaked and single-crossing preference profiles which are *minimally not* one-dimensional Euclidean, i.e., excluding any single voter from each of these profiles makes it one-dimensional Euclidean.

We refer to the work of Bredereck et al. (2016) and Elkind et al. (2017) and the literature cited there for further discussion of the single-peaked, the single-crossing, and the one-dimensional Euclidean preference domains.

**Our contribution.** Recently, Chen et al. (2017) provided a single-peaked and single-crossing profile with three voters and six alternatives which is not one-dimensional Euclidean. In this paper, we show that their counterexample is indeed minimal in terms of the number of voters and the number of alternatives.

In terms of the number of voters, we show that any two single-peaked preference orders are always one-dimensional Euclidean. One way to achieve this would have been to analyze the linear inequalities induced by the linear programs (LP) for detecting one-dimensional Euclideanness (Doignon and Falmagne 1994; Knoblauch 2010; Elkind and Faliszewski 2014). We chose, however, to provide a direct algorithm that, given a single-peaked preference profile with two voters, constructs a concrete one-dimensional Euclidean embedding (see Algorithm 1). The reason for this choice is three-fold.

- First, from a social-choice and mathematical point of view, through our approach we not only know that all two-voter single-peaked preference profiles are one-dimensional Euclidean but also know that there are one-dimensional Euclidean embeddings of all such profiles which display a certain uniform structure. Note that if we had chosen to analyze the corresponding linear program and tried to show that it is always feasible, we may not be able to have a visual understanding of what the embeddings look like unless we had used an LP solver to solve the induced linear inequalities. Moreover, the embeddings provided by an LP solver may have looked completely different between different profiles.
- Second, from an algorithmic point of view, our approach is constructive and does not need any external LP solver. The algorithm is so simple that we can provide a publicly available software for individual checking.
- Third, from a complexity point of view, our algorithm is faster than the LP-solver approach. For *m* alternatives, our algorithm runs in $O(m\cdot \mathsf {runtime}\text {-}\mathsf {mult}(m))$ time, where $\mathsf {runtime}\text {-}\mathsf {mult}(m)$ denotes the running time of the multiplication of two integer numbers of *O*(*m*) bits each, while the LP-solver approach would need to check the feasibility of the underlying LP formulation in time $O(\log ^3(m)\cdot \mathsf {runtime}\text {-}\mathsf {matrix}(m))$ (van den Brand 2020), where $\mathsf {runtime}\text {-}\mathsf {matrix}(m)$ denotes the running time of multiplying an $m\times m$ dimensional matrix. The multiplication of two integers of *O*(*m*) bits can be done in time $\mathsf {runtime}\text {-}\mathsf {mult}(m)=O(m\cdot \log (m)\cdot \log (\log (m)))$ (Schönhage and Strassen 1971) while the fastest known algorithm for $m\times m$-dimensional matrix multiplication runs in time $\mathsf {runtime}\text {-}\mathsf {matrix}(m) =O(m^{2.38})$ (Gall 2014).

As for the number of alternatives, we use the constraint solver CPLEX^1^ to show that all single-peaked and single-crossing preferences with up to five alternatives are one-dimensional Euclidean (see Theorem 3). The proof for the second result is computer-aided and can be verified online (see Sect. 4). We note that we did not use the approach given by Doignon and Falmagne (1994), Knoblauch (2010), and Elkind and Faliszewski (2014) to first calculate a linear order of the alternatives and the voters that is consistent to a one-dimensional Euclidean embedding. Instead, our CPLEX program is simply a direct translation of the one-dimensional Euclidean constraints on the variables representing the alternatives and the voters (see Definition 3). The reason for this is that the CPLEX solver offers a function of returning the absolute value of the difference of any two variables, so a linear order of the alternatives and the voters is not necessary to formulate the one-dimensional Euclidean constraints in CPLEX. The verification that every computed embedding is indeed one-dimensional Euclidean is done in a straightforward way, namely by going through each voter’ preference order and comparing the distances between the voter’s and alternatives’ embedded positions.

Identifying the smallest single-peaked and single-crossing preference profile that is not one-dimensional Euclidean not only helps to better understand what precludes a preference profile from being one-dimensional Euclidean, but can also be seen as a necessary step on the way towards a compact characterization via forbidden, albeit not finitely many, subprofiles. We remark that compact characterization via infinitely many forbidden substructures has been done for mathematical concepts such as interval graphs (Lekkerkerker and Boland 1962) and the consecutive ones property in binary matrices (Tucker 1972).

**Paper outline.** In Sect. 2, we introduce necessary definitions, including single-peaked and single-crossing preferences, and one-dimensional Euclidean preferences. We also discuss some fundamental observations regarding these domain restrictions. In Sect. 3, we formulate our first main result in Theorems 1 and 2. We prove this result by providing a simple and direct algorithm (see Algorithm 1) that constructs a one-dimensional Euclidean embedding for any two preference orders which are single-peaked. We also provide an example to illustrate Algorithm 1 (see Example 3). In Sect. 4, we provide our second main result by describing the computer program that finds all possible preference profiles with up to five alternatives that are both single-peaked and single-crossing, and uses the CPLEX solver publicly available for researchers to provide a one-dimensional Euclidean embedding for each of these profiles (see Theorem 3). Both the source and the embeddings for all produced profiles (including the verification) are available online from https://owncloud.tuwien.ac.at/index.php/s/nysw13YkUajJpOn and https://owncloud.tuwien.ac.at/index.php/s/Pk8TZxva48LJt35, respectively. For the sake of readability, the proofs of lemmas marked with an asterisk ($*$) have been moved to the appendix.

## Definitions and notations

Let ${{\mathcal {A}}}:=\{1,\ldots ,m\}$ be a set of alternatives. A *preference order* $\succ$ of ${{\mathcal {A}}}$ is a linear order of ${{\mathcal {A}}}$; a linear order is a binary relation which is total, irreflexive, asymmetric, and transitive. For two distinct alternatives *a* and *b*, the relation $a\succ b$ means that *a* is strictly preferred to (or in other words, ranked higher than) *b* in $\succ$. An alternative *c* is *the most-preferred alternative in* $\succ$ if for each alternative $b\in {{\mathcal {A}}}\setminus \{c\}$ it holds that $c \succ b$.

Let $\succ$ be a preference order over ${{\mathcal {A}}}$. We use $\succeq$ to denote the binary relation which includes $\succ$ and preserves the reflexivity, i.e., $\succeq \, := \succ \cup \{(a,a)\mid a\in {{\mathcal {A}}}\}$. For a subset *B* of alternatives and an alternative *c* not in *B*, we use $B\succ c$ to refer that each $b\in B$ is preferred to *c* in $\succ$.

A *preference profile* ${{\mathcal {P}}}$ specifies the preference orders of some voters over some alternatives. Formally, ${{\mathcal {P}}} := ({{\mathcal {A}}}, {{\mathcal {V}}}, {{\mathcal {R}}}:=(\succ _1, \ldots , \succ _n))$, where ${{\mathcal {A}}}$ denotes the set of *m* alternatives, ${{\mathcal {V}}}$ denotes the set of *n* voters, and ${{\mathcal {R}}}$ is a collection of *n* preference orders such that each voter $v_i\in {{\mathcal {V}}}$ ranks the alternatives according to the preference order $\succ _i$ on ${{\mathcal {A}}}$. We also assume that no two voters in a preference profile have the same preference order.

### Single-peaked preferences

The single-peaked property was introduced by Black (1958) and has since been studied extensively.

#### Definition 1

(*single-peakedness*) A preference order $\succ$ on a set ${{\mathcal {A}}}$ of alternatives is *single-peaked* with respect to a linear order $\rhd$ of ${{\mathcal {A}}}$ if for each two distinct alternatives $b,c \in {{\mathcal {A}}}\setminus \{a^*\}$ it holds that

$$\begin{aligned} \text { if } c \rhd b \rhd a^* \text { or } a^*\rhd b \rhd c, \text {then } b \succ c, \end{aligned}$$

where $a^*$ is the most-preferred alternative in $\succ$.

A preference profile ${{\mathcal {P}}}=({{\mathcal {A}}},{{\mathcal {V}}},{{\mathcal {R}}})$ is *single-peaked* if there is a linear order $\rhd$ of the alternatives ${{\mathcal {A}}}$ such that the preference order of each voter from ${{\mathcal {V}}}$ is single-peaked with respect to $\rhd$.

Slightly abusing the terminology, we say that two preference orders are *single-peaked* if there is a linear order with respect to which each of these two preference orders is single-peaked.

The single-peaked property can be characterized by two forbidden subprofiles, worst-diverse configurations and $\alpha$-configurations (Ballester and Haeringer 2011). The former is defined on three preference orders while the latter is defined on two preference orders. For *two* arbitrary preference orders, this means that no $\alpha$-configurations are present, implying the following.

#### Proposition 1

(Ballester and Haeringer 2011) *Two preference orders, denoted as* $\succ _1$ *and* $\succ _2$, *over the set* ${{\mathcal {A}}}$ *of alternatives are single-peaked if and only if for all four distinct alternatives* $u,v,w,z\in {{\mathcal {A}}}$ *such that* $u\succ _1 v \succ _1 w$ *and* $w\succ _2 v \succ _2 u$ *it holds that* $v \succ _1 z$ or $v \succ _2 z$.

### Single-crossing preferences

The concept of single-crossing profiles dates back to the seventies, when Mirrlees (1971) and Roberts (1977) observed that voters voting on income taxation may form a linear order such that between each two tax rates, the voters that have the same opinion on the relative positions of both rates are either on the top or at the bottom of the linear order.

#### Definition 2

(*single-crossingness*) Given a preference profile ${{\mathcal {P}}}=({{\mathcal {A}}},{{\mathcal {V}}},{{\mathcal {R}}})$, a linear order $\rhd$ of the voters ${{\mathcal {V}}}$ is *single-crossing with respect to a pair* $\{a,b\}\subseteq {{\mathcal {A}}}$ of alternatives if the set $\{v_i \in {{\mathcal {V}}}\mid a \succ _i b\}$ is an interval in $\rhd$. It is a single-crossing order for ${{\mathcal {P}}}$ if it is single-crossing with respect to every pair of alternatives.

Preference profile ${{\mathcal {P}}}$ is *single-crossing* if it admits a single-crossing order of the voters.

The single-crossing property can be characterized by two forbidden subprofiles, $\gamma$-configurations and $\delta$-configurations (Bredereck et al. 2013).

### One-dimensional Euclidean preferences

#### Definition 3

(*one-dimensional Euclideanness*) Let ${{\mathcal {P}}}:=({{\mathcal {A}}}, {{\mathcal {V}}}:=\{v_1, \ldots , v_n\}, {{\mathcal {R}}}:=(\succ _1, \ldots , \succ _n))$ be a preference profile. Let $E:{{\mathcal {A}}}\cup V \rightarrow {\mathbb {R}}$ be an embedding of the alternatives and the voters into the real line where each two distinct alternatives $a, b\in {{\mathcal {A}}}$ have different values, that is, $E(a)\ne E(b)$. A voter $v_i \in V$ is *one-dimensional Euclidean with respect to* *E* if for each two distinct alternatives $a, b \in {{\mathcal {A}}}$ voter $v_i$ strictly prefers the one closer to him, that is,

$$\begin{aligned} a \succ _i b \quad \text { if and only if } |E(a) - E(v_i)| < |E(b) - E(v_i)|. \end{aligned}$$

An embedding *E* of the alternatives and voters is a *one-dimensional Euclidean embedding* of profile ${{\mathcal {P}}}$ if each voter in *V* is one-dimensional Euclidean with respect to *E*.

A preference profile is *one-dimensional Euclidean* if it admits a one-dimensional Euclidean embedding.

Example 1 illustrates the concepts of single-peaked, single-crossing, and one-dimensional Euclidean preferences.

#### Example 1

Consider the following preference profile with five alternatives $\{1,2,3,4,5\}$ and six voters $v_1,v_2,\ldots ,v_6$.

$$\begin{aligned} &v_1\!:\!1 \succ _1 2 \succ _1 3 \succ _1 4 \succ _1 5,\\ & v_2\!:\!3 \succ _2 1 \succ _2 2 \succ _2 4 \succ _2 5,\\ &v_3\!:\!2 \succ _3 1 \succ _3 4 \succ _3 5 \succ _3 3,\\ & v_4\!:\!2 \succ _4 4 \succ _4 1 \succ _4 5 \succ _4 3,\\ & v_5\!:\!4 \succ _5 2 \succ _5 5 \succ _5 1 \succ _5 3,\\ &v_6\!:\!5 \succ _6 4 \succ _6 2 \succ _6 1 \succ _6 3. \end{aligned}$$

It admits exactly two single-peaked orders, namely $5 \rhd 4 \rhd 2 \rhd 1 \rhd 3$ and the reverse of $\rhd$. The single-crossing order of the profile is *v*_6_ ▸ *v*_5_ ▸ *v*_4_ ▸ *v*_3_ ▸ *v*_1_ ▸ *v*_2_ and the reverse of ▸. Note that, in contrast to the single-peaked order, the single-crossing order is unique up to reversal.

The profile is also one-dimensional Euclidean, as shown by the following one-dimensional Euclidean embedding. 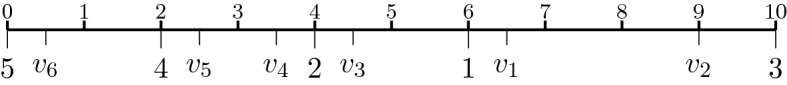


The following observation regarding the relation between single-peaked and single-crossing profiles and the one-dimensional Euclidean representation is also known from the literature (Coombs 1964; Doignon and Falmagne 1994; Chen et al. 2017).

#### Observation 1

*If a profile is* one-dimensional Euclidean, *then it is also single-peaked and single-crossing.*

#### Proof

It is straightforward to see that if there is a one-dimensional Euclidean representation *E* of a given profile, then this profile is single-peaked with respect to the order induced by ordering the alternatives increasingly (or decreasingly) according to their values in *E*. Moreover, it is single-crossing with respect to the order induced by ordering the voters increasingly (or decreasingly) according to their values in *E*. $\square$

## Single-peaked preference profiles with two voters

In this section, we formulate and prove our first main result.

### Theorem 1

*A profile* $\mathcal {P}$ *with two voters is one-dimensional Euclidean if and only if it is single-peaked.*

### Proof

The “only if” part follows from Observation 1. The “if” part follows from Theorem 2. $\square$

To complete the proof of Theorem 1, we show the following.

### Theorem 2

*Given a single-peaked preference profile with two voters and* *m* *alternatives as input, Algorithm 1 returns a* one-dimensional Euclidean *embedding of the profile in* $O(m\cdot \mathsf {runtime}\text {-}\mathsf {mult}(m))$ *time, where* $\mathsf {runtime}\text {-}\mathsf {mult}(m)$ *denotes the running time of the multiplication of two integers of* *O*(*m*) *bits each.*^2^

In the following, in Sect. 3.1, we first present Algorithm 1 and observe some of its technical properties, and in Sect. 3.3 we prove Theorem 2.

### Algorithm 1 and some technical results

Our algorithm for Theorem 2 (see 1D-Euclid-Embed($\succ _1,\succ _2$) in Algorithm 1) is an “inside-out” approach and comprises two stages, an initialization stage (lines 2–9) and an iteration stage (lines 10–13).

In the initialization stage, we embed all *inner* alternatives (see Definition 4) that are ranked by both voters $v_1$ and $v_2$ between their respective most-preferred alternatives, denoted as $a_1$ and $b_1$ throughout the whole section; note that $a_1$ can be equal to $b_1$. After that we embed voter $v_1$ to the right of, and voter $v_2$ to the left of, the inner alternatives which we have just embedded.

**Fig. 1:**
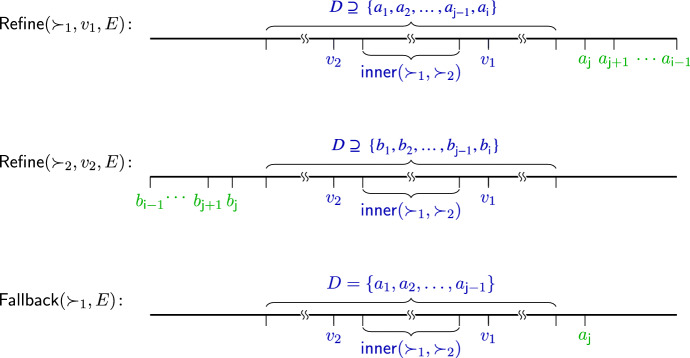
Illustration of the three possible procedures called in lines 11–13 of Algorithm 1. *D* denotes the set of embedded alternatives right before the call of each procedure. The two sets, $\{{a_{{j}}, a_{{ {j}}+1}},\ldots , a_{ {i-1}}\}$ and $\{{b_{{j}}, b_{{ {j}}+1}},\ldots , b_{{i-1}}\}$, denote the sets of alternatives that are to be embedded in a call to Refine($\succ _1,v_1,E$) and Refine($\succ _2,v_2,E$), respectively. In Fallback($\succ _1,E$), only $a_{{\mathsf {j}}}$ will be embedded

In the iteration stage, we iterate over the remaining alternatives, in order of the preferences of each voter, and try to embed them to the right part of voter $v_1$ or to the left part of voter $v_2$. More specifically, we repeatedly call Refine() to find a range of alternatives and embed them either to the right of the right-most alternative or to the left of the left-most alternative in the embedding. The alternatives are those which are preferred (either by voter $v_1$ or voter $v_2$) to some already embedded alternatives. If no such alternatives exist, we call Fallback() to find a next alternative less preferred by voter $v_1$ and embed it to the right of the right-most alternative. See Fig. 1 for an illustration. The single-peaked property, according to Proposition 1, guarantees that the newly embedded alternatives (through either Refine or Fallback) do not alter the one-dimensional Euclidean property. 
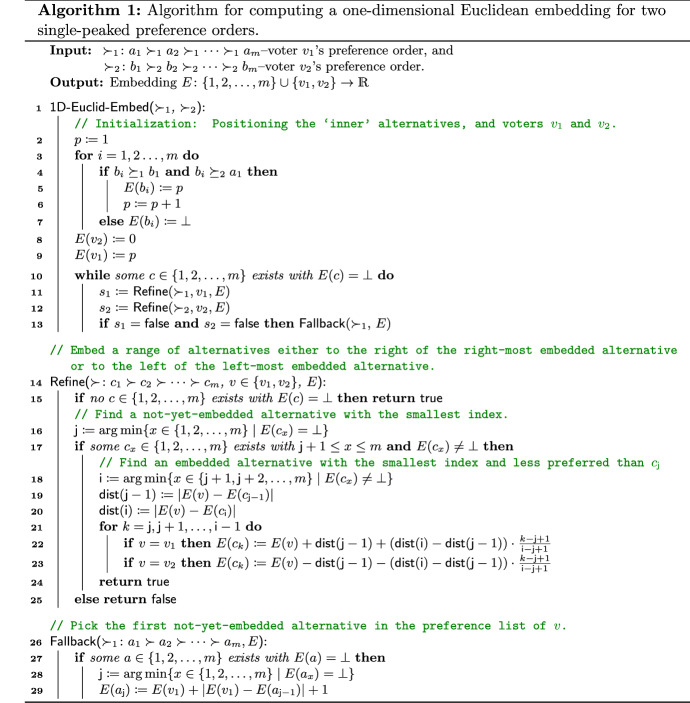

#### Initialization (lines 2–9 of Algorithm 1)

To describe the initialization stage in more detail, we introduce the notion of inner alternatives.

##### Definition 4

(*Inner alternatives*) Let $\succ _1$ and $\succ _2$ be two preference orders, and let $a_1$ and $b_1$ be the most-preferred alternatives of $\succ _1$ and $\succ _2$, respectively. The *set of inner alternatives of* $\succ _1$ and $\succ _2$, denoted as $\mathsf {inner}(\succ _1, \succ _2)$, is the set of all alternatives that are ranked between $a_1$ and $b_1$ by both $\succ _1$ and $\succ _2:$

$$\begin{aligned} \mathsf {inner}(\succ _1,\succ _2):=\{c \in \{1,2,\ldots ,m\} \mid c \succeq _1 b_1 \wedge c \succeq _2 a_1\} \text {.} \end{aligned}$$

##### Example 2

Consider two preference orders $$\succ _1$$ and $$\succ _2$$ with $$1\succ _1 2 \succ _1 3 \succ _1 4$$ and $$3 \succ _2 4 \succ _2 2 \succ _2 1$$. The set of inner alternatives of $$\succ _1$$ and $$\succ _2$$ is $$\mathsf {inner}(\succ _1, \succ _2)=\{1,2,3\}.$$$$\square $$

We observe the following properties concerning the inner alternatives of two single-peaked preference orders.

##### Lemma 1

*Consider two preference orders* $\succ _1$ *and* $\succ _2$.

1. *For each* $r\in \{1,2\}$, *the most-preferred alternative of* $\succ _r$ *belongs to* $\mathsf {inner}(\succ _1,\succ _2)$.
2. *If* $\succ _1$ *and* $\succ _2$ *are single-peaked, then for each two distinct inner alternatives* $x, y\in \mathsf {inner}(\succ _1,\succ _2)$ *it holds that* $x\succ _1 y$ *if and only if* $y \succ _2 x$.

##### Proof

The first statement follows from the definition of $\mathsf {inner}(\succ _1,\succ _2)$.

It remains to show Statement (2). If $a_1=b_1$, then by the definition of $\mathsf {inner}$ it holds that $\mathsf {inner}(\succ _1,\succ _2)=\{a_1\}=\{b_1\}$, and the second statement holds immediately since $\mathsf {inner}(\succ _1,\succ _2)$ has only one alternative. Thus, let us assume that $a_1 \ne b_1$ so that $|\mathsf {inner}(\succ _1,\succ _2)|\ge 2$. Let $\succ _1$ and $\succ _2$ be single-peaked. Suppose, for the sake of contradiction, that there are two distinct alternatives $x,y\in \mathsf {inner}(\succ _1,\succ _2)$ with $x\succ _1 y$ and $x \succ _2 y$—the case with $y \succ _1 x$ and $y \succ _2 x$ works analogously. Since $a_1\succ _1 b_1$ and $b_1 \succ _2 a_1$, it follows that $x,y \notin \{a_1,b_1\}$. By the definition of $a_1$ and $b_1$ and since $x,y\in \mathsf {inner}(\succ _1,\succ _2)$, this implies that $a_1 \succ _1 x \succ _1 y \succ _1 b_1$ and $b_1 \succ _2 x \succ _2 y \succ _2 a_1$—a contradiction to Proposition 1. $\square$

Now, we are ready to describe the initialization stage of Algorithm 1, where we first embed all inner alternatives (lines 2–7). By Proposition 1, voters $v_1$ and $v_2$ have exactly the opposite preferences with respect to these inner alternatives. Hence, by the single-peaked property, the order of the inner alternatives induced by any one-dimensional Euclidean embedding is fixed (up to reverse). In other words, the induced order corresponds to the preferences of either voter $v_1$ or voter $v_2$. Without loss of generality, we choose the induced order to correspond to the preferences of voter $v_2$. More precisely, if the preference order of $v_2$ restricted to the inner alternatives is $c_1\succ c_2\succ \cdots \succ c_x$, where $x=|\mathsf {inner}(\succ _1,\succ _2)|$, then we let each two consecutive alternatives in $\succ$ have unit distance. Then, in lines 8–9 we embed voter $v_2$ to the left of $c_1$ and $v_1$ to the right of $c_x$, again at unit distance.

Summarizing, we observe the following about the initialization stage.

##### Proposition 2

*Let* *E* *be the embedding constructed by the end of the initialization phase (lines 2–9) of Algorithm 1. Let* $c_1, c_2, \ldots , c_x$ *be the embedded alternatives with* $E(c_1)<\cdots < E(c_x)$. *The following holds.*

1. $$\mathsf {inner}(\succ _1,\succ _2)=\{c_1,c_2,\dots ,c_x\}$$ with $$c_x=a_1$$ and $$c_1=b_1$$.
2. *The preferences of voter* $v_2$ *restricted to* $\mathsf {inner}(\succ _1,\succ _2)$ are $c_1 \succ _2 c_2 \succ _2 \dots \succ _2 c_x$.
3. $$E(v_1)-E(v_2)=|\mathsf {inner}(\succ _1,\succ _2)|+1$$.
4. $$E(v_2) < E(c_1)$$ *and* $$E(c_{x})<E(v_1)$$.
5. *If* $\succ _1$ *and* $\succ _2$ *are single-peaked, then the preferences of voter* $v_1$ *restricted to* $\mathsf {inner}(\succ _1,\succ _2)$ are $c_x \succ _1 c_{x-1} \succ _1 \dots \succ _1 c_1$.

##### Proof

The first three statements follow directly from lines 3–7 and from the definition of $\mathsf {inner}(\succ _1,\succ _2)$. Moreover, it holds that $E(c_1)=1$, $E(c_x)=|\mathsf {inner}(\succ _1,\succ _2)|$, $E(v_2)=0$, and $E(v_1)=|\mathsf {inner}(\succ _1,\succ _2)|+1$. This implies Statement 4.

As to Statement 5, consider an arbitrary embedded alternative $c_j$ with $j\in \{1,\ldots ,x-1\}$. Then, by the second statement, we have that $c_j \succ _2 c_{j+1}$. By Lemma 1(2), we have that $c_{j+1} \succ _1 c_j$. $\square$

#### The iteration stage (lines 10–13 of Algorithm 1)

After having embedded all inner alternatives and the two voters, the main loop (lines 10–13) extends the embedding by alternatingly placing alternatives that should be embedded to the right of the right-most embedded alternative and alternatives that should be embedded to the left of the left-most embedded alternative. The corresponding procedure is called Refine() (lines 14–25) and is used for both voters $v_1$ and $v_2$. It searches through the alternatives along the preference order of $v_1$ (resp. $v_2$), and finds all not-yet-embedded alternative(s) *C* which $v_1$ (resp. $v_2$) ranks between two already embedded alternatives. To make sure the other not-yet-embedded alternatives can still be embedded at a later stage, we embed the found alternatives *C* to the right (resp. left) of the right-most (resp. left-most) embedded alternative. The Fallback($\succ _1,E$) procedure in line 13 guarantees that at least one alternative is embedded during each iteration, thus ensuring that the algorithm terminates.

For an illustration, see the following example.

**Table 1:**
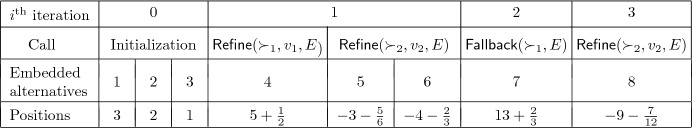
A summary of how Algorithm 1 proceeds for an input of the two preference orders $\succ _1$ and $\succ _2$ given in Example 3. The first row refers to the $i{\text {th}}$ iteration in the main loop, where $i=0$ refers to the initialization. The second row shows exactly which procedure is called with which arguments. The third and the last rows show which alternatives are embedded at which positions in that call

##### Example 3

(Illustration for Algorithm 1) Consider the following preference profile with two voters and eight alternatives:

$$\begin{aligned}&  v_1\! :\!1 \succ _1 4 \succ _1 2 \succ _1 3 \succ _1 5 \succ _1 6 \succ _1 7 \succ _1 8,\\ & v_2\! :\!3 \succ _2 2 \succ _2 1 \succ _2 5 \succ _2 6 \succ _2 4 \succ _2 8 \succ _2 7. \end{aligned}$$

It is single-peaked with respect to the order $\rhd$ with $8 \rhd 6 \rhd 5 \rhd 3 \rhd 2 \rhd 1 \rhd 4 \rhd 7$, and also with respect to the reverse of $\rhd$. Given the two preferences orders as input, our algorithm will return a one-dimensional Euclidean embedding which is depicted in the following line. 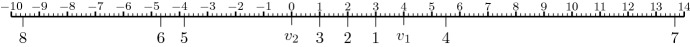


Table 1 summarizes how the algorithm proceeds with $v_1$ and $v_2$ as input.

More precisely, in the initialization, the inner alternatives $\mathsf {inner}(\succ _1,\succ _2)=\{1,2,3\}$ are embedded between voter $v_1$ at 4 and voter $v_2$ at 0.

In iteration 1 of the main loop (lines 10–13), alternative 4 is embedded to the right of voter $v_1$ in the first call to Refine($\succ _1,v_1,E$), as it is the first not-yet-embedded alternative in the preferences of $v_1$. Since $c_2=4$, we set ${\mathsf {j}}:=2$ in line 16. Since $c_3=2$ is an embedded alternative with the smallest index which $v_1$ ranks lower than 4, we set ${\mathsf {i}}:=3$ in line 18. Next, we set $\textsf {dist}({\mathsf {j}}-1):=|E(v_1)-E(c_{{\mathsf {j}}-1})| = |4-3| = 1$ and $\textsf {dist}({\mathsf {i}}) :=|E(v_1)-E(c_{{\mathsf {i}}})| = |4-2| = 2$ in lines 19–20. Finally, for $k={\mathsf {j}}=2$ we set

$$\begin{aligned} E(4) = E(c_2) = E(c_k)&:=E(v_1) + \textsf {dist}({\mathsf {j}}-1) + (\textsf {dist}({\mathsf {i}}) - \textsf {dist}({\mathsf {j}}-1))\cdot \frac{k-{\mathsf {j}}+1}{{\mathsf {i}}-{\mathsf {j}}+1}\\&= 4 + 1 + (2-1)\cdot \frac{1}{2} = 5+\frac{1}{2}. \end{aligned}$$

After alternative 4 has been embedded, alternatives 5 and 6 are embedded to the left of the left-most alternative, namely 3, in the first call to Refine($\succ _2,v_2,E$).

This is because alternatives 5 and 6 are the first not-yet-embedded alternatives in the preference order of $v_2$, and there is an embedded alternative, namely 4, such that $v_2$ prefers $\{5,6\}$ to 4. So, ${\mathsf {j}}:=4$ and ${\mathsf {i}}:=6$, $\textsf {dist}({\mathsf {j}}-1) :=3$, and $\textsf {dist}({\mathsf {i}})=5+\frac{1}{2}$. Hence, for $k={\mathsf {j}}=4$,

$$\begin{aligned} E(5) = E(c_4) = E(c_k)&:=E(v_2) - \textsf {dist}({\mathsf {j}}-1) - (\textsf {dist}({\mathsf {i}}) - \textsf {dist}({\mathsf {j}}-1))\cdot \frac{k-{\mathsf {j}}+1}{{\mathsf {i}}-{\mathsf {j}}+1}\\&= 0 - 3 - \left( 5+\frac{1}{2}-3\right) \cdot \frac{1}{3} = -3-\frac{5}{6}. \end{aligned}$$

For $$k={\mathsf {j}}+1=5$$,

$$\begin{aligned} E(6) = E(c_5) = E(c_k)&:=E(v_2) - \textsf {dist}({\mathsf {j}}-1) - (\textsf {dist}({\mathsf {i}}) - \textsf {dist}({\mathsf {j}}-1))\cdot \frac{k-{\mathsf {j}}+1}{{\mathsf {i}}-{\mathsf {j}}+1}\\&= 0 - 3 - \left( 5+\frac{1}{2}-3\right) \cdot \frac{2}{3} = -4-\frac{2}{3}. \end{aligned}$$

Fallback($\succ _1,E$) is not called since at least one of the calls to Refine() returned $\mathsf {true}$.

In iteration 2, that is, after alternatives 5 and 6 have been embedded, neither Refine($\succ _1, v_1, E$) nor Refine($\succ _2,v_2,E$) return $\mathsf {true}$. Alternative 7 is the first not-yet-embedded alternative in the preference order of $v_1$. Thus, Fallback($\succ _1,E$) embeds 7 to the right of $v_1$ so that it becomes the right-most alternative with ${\mathsf {j}}:=7$:

$$\begin{aligned} E(7)=E(a_{\mathsf {j}})=E(a_7)&:=E(v_1) + |E(v_1)-E(a_{{\mathsf {j}}-1})| + 1 = 13+\frac{2}{3}. \end{aligned}$$

At last, in iteration 3, Refine($\succ _1,v_1,E$) returns $\mathsf {false}$. Then, in Refine($\succ _2, v_2, E$), alternative 8 is embedded to the left of the left-most alternative, namely 6, as 8 is the first not-yet-embedded alternative in the preferences of $v_2$ and there is an embedded alternative, namely 7, such that $v_2$ prefers alternative 8 to 7. So, ${\mathsf {j}}:=7$ and ${\mathsf {i}}:=8$, $\textsf {dist}({\mathsf {j}}-1) :=5+\frac{1}{2}$, and $\textsf {dist}({\mathsf {i}})=13+\frac{2}{3}$. For $k={\mathsf {j}}=7$,

$$\begin{aligned} E(8) = E(c_k) = E(c_7)&:=E(v_2) - \textsf {dist}({\mathsf {j}}-1) - (\textsf {dist}({\mathsf {i}}) - \textsf {dist}({\mathsf {j}}-1))\cdot \frac{k-{\mathsf {j}}+1}{{\mathsf {i}}-{\mathsf {j}}+1}\\&= 0 - \left( 5+\frac{1}{2}\right) - \left( 13+\frac{2}{3}-5-\frac{1}{2}\right) \cdot \frac{1}{2} = -9-\frac{7}{12}.&~~\qquad \qquad \end{aligned}$$

$$\square $$

Due to the way we embed the alternatives in Refine($\succ ,v,E$), the newly embedded alternatives do not violate the one-dimensional Euclidean property for voter *v*, as stated in the following lemma.

##### Lemma 2

($*$) *Let* ${\mathsf {j}}$ *and* ${\mathsf {i}}$ *be as defined in a call to* Refine($\succ _z,v_z, E$) *with* $z\in \{1,2\}$. *If* *E* *satisfies* $|E(c_{{\mathsf {j}}-1})-E(v_z)| < |E(c_{{\mathsf {i}}})-E(v_z)|$, *then after the call to* Refine($\succ _z,v_z,E$) *it holds that* $|E(c_{{\mathsf {j}}-1})-E(v_z)|<|E(c_{\mathsf {j}})-E(v_z)|<\dots< |E(c_{{\mathsf {i}}-1})-E(v_z)|<|E(c_{{\mathsf {i}}})-E(v_z)|$.

### Running time of Algorithm 1

We close this subsection by analyzing the running time of Algorithm 1. Clearly, the initialization in lines 2–9 can be done in *O*(*m*) time. Recall that the number of alternatives embedded during the initialization phase is ${p-1}$. Then, the main loop in lines 10–13 terminates after at most $m-p+1$ iterations since in each iteration at least one of the calls to Refine($\succ _z,v_z,E$) with $z\in \{1,2\}$ or Fallback($\succ _1,E$) will embed at least one more alternative.

Hence, it remains to analyze the running time of embedding the alternatives via a call to Refine or Fallback. Since each such alternative is embedded exactly once, which needs a constant number of multiplications, additions, and subtractions (see lines 19–23 and line 29), we only need to analyze the running time of the multiplication in the assignments in lines 22–23, as well as the running time of finding the two indices ${\mathsf {j}}$ and ${\mathsf {i}}$ in lines 16, 18, and 28.

**Implementation for finding** ${\mathsf {j}}$ **and** ${\mathsf {i}}$. A straightforward implementation may require $O(m^2)$ time in total to find ${\mathsf {j}}$ and ${\mathsf {i}}$ in all calls to Refine($\succ _z,v_z,E$) and Fallback($\succ _1,E$). However, with a few additional variables we can find them in *O*(*m*) time in total in all calls. For brevity’s sake, we have left this optimization out of the description of Algorithm 1. A modified version that includes it can be found in Appendix B.1.

Next, we analyze the running time for a possible multiplication in lines 22–23 of Algorithm 2. To this end, let us assume that we store the position of each embedded alternative $c_i$ in a integer triple $(e_i,f_i,g_i)$ such that $E(c_i)=e_i+\frac{f_i}{g_i}$ with $e_i,f_i,g_i \in \mathbb {Z}$ and $f_i < g_i$. Then, a multiplication in lines 22–23 can be done via integer multiplication, whose running time depends on the largest $|e_i|+1$ and the largest *denominator* $g_i$ in the triple encoding.

**Largest value computed.** To estimate the largest value, we only need to estimate the largest distance from either voter to all alternatives. Observe that in Refine($\succ _z,v_z,E$), since the distance from $v_z$ to each of the newly embedded alternatives is smaller than the distance from $v_z$ to $c_{{\mathsf {i}}}$ (which is already embedded), the largest distance from $v_z$ to any embedded alternative is not increased. The distance from $v_{3-z}$ to each of the newly embedded alternatives could be enlarged by *p*, which is the distance between the two voters. The same holds for Fallback($\succ _1,E$). Hence, the largest distance from either voter to all alternatives is bounded by $p\cdot (m-p+1) \in O(m^2)$.

**Largest denominator computed.** As for the largest computed denominator, we observe that it depends on the number of alternatives to be embedded. To this end, let $x_1,x_2,\ldots ,x_{n}$, $n\le m-p+1$ denote the number of alternatives embedded in a successful call of Refine $(\succ ,v,E)$. Then, the largest computed denominator is bounded by the following:

$$\begin{aligned} \prod _{z=1}^{n}(x_i+1) \le \Bigg (\frac{\sum _{z=1}^{n}x_z+1}{n}\Bigg )^n \le&\Bigg (\frac{m-p+1+n}{n}\Bigg )^n\\ {\mathop {\le }\limits ^{\text {let } n\;:=\;\alpha \cdot (m-p+1)}}&\Big (1+\frac{1}{\alpha }\Big )^{\alpha \cdot (m-p+1)} \le e^{m-p+1} \le e^m, \end{aligned}$$

where the first inequality is due to inequality of arithmetic and geometric means, the second inequality is due to the fact that the total number of alternatives embedded during the iteration stage is bounded by $m-{p-1}$, the fourth inequality is due to the fact that $n \le m-{p-1}$, and *e* denotes the Euler number.

Since the multiplication of two integers of value $O(e^m)$ can be done in $O(m\cdot \log (m) \cdot \log (\log (m)))$ time (Schönhage and Strassen 1971) and since for each embedded alternative our algorithm performs a constant number of integer multiplications, additions, and subtractions, our algorithm can be implemented to run in $O(m^2\cdot \log (m) \cdot \log (\log (n)))$ time.

### Proof of Theorem 2

In Sect. 3.2, we prove the running time stated in Theorem 2. To prove Theorem 2, it remains to prove that Algorithm 1 is correct, i.e., it returns a one-dimensional Euclidean embedding whenever the two input preference orders are single-peaked. To this end, let $\succ _1$ and $\succ _2$ be the input reference orders with $\succ _1:a_1\succ _1 a_2 \succ _1 \cdots \succ _1 a_m$ and $\succ _2:b_1\succ _2 b_2\succ _2 \cdots \succ _2 b_m$. First of all, by Proposition 2, the initialization phase computes a one-dimensional Euclidean embedding of $\succ _1$ and $\succ _2$ when restricted to the inner alternatives $\mathsf {inner}(\succ _1, \succ _2)$. Thus, to prove the correctness, we only need to show that each iteration of the main loop (lines 10–13) extends the embedding in such a way that it is one-dimensional Euclidean for $\succ _1$ and $\succ _2$ when restricted to the embedded alternatives. To achieve this, we need to show that the procedures Refine($\succ _z,v_z,E$), $z\in \{1,2\}$, and Fallback($\succ _1,E$), extend the existing one-dimensional Euclidean embedding to one that is one-dimensional Euclidean with respect to the alternatives that have already been embedded and those which are newly embedded. To this end, we introduce a few more technical notions regarding a subset of alternatives.

#### Definition 5

Let $E:{{\mathcal {A}}}\cup \{v_1,v_2\}\rightarrow {\mathbb {R}}\cup \{\bot \}$ be an embedding of a two-voter preference profile ${{\mathcal {P}}}=({{\mathcal {A}}},{{\mathcal {V}}},{{\mathcal {R}}})$ with ${{\mathcal {V}}}=\{v_1,v_2\}$ and ${{\mathcal {R}}}=(\succ _1,\succ _2)$. Let ${{\mathcal {A}}}'\subseteq {{\mathcal {A}}}$ be a non-empty subset of alternatives. We say that *E* is *one-dimensional Euclidean with respect to* ${{\mathcal {A}}}'$ if for each voter $v_z\in {{\mathcal {V}}}$ and each two alternatives $x, y \in {{\mathcal {A}}}'$ it holds that $E(x)\ne \bot$, $E(y)\ne \bot$ and

$$\begin{aligned} x \succ _z y \text { if and only if } |E(x) - E(v_z)| < |E(y) - E(v_z)|. \end{aligned}$$

For each voter $v_z\in {{\mathcal {V}}}$, we use $\mathsf {worst}({{\mathcal {A}}}',v_z)$ to denote the alternative from ${{\mathcal {A}}}'$ which is least preferred by $v_z$, i.e.,

$$\begin{aligned} \mathsf {worst}({{\mathcal {A}}}',v_z) \in {{\mathcal {A}}}' \text { such that } \forall x\in {{\mathcal {A}}}'\text { it holds that } x\succeq _z \mathsf {worst}({{\mathcal {A}}}',v_z). \end{aligned}$$

#### Example 4

Consider the profile from Example 3 again.

$$\begin{aligned} &v_1 \!:\!1 \succ _1 4 \succ _1 2 \succ _1 3 \succ _1 5 \succ _1 6 \succ _1 7 \succ _1 8,\\ & v_2\! :\!3 \succ _2 2 \succ _2 1 \succ _2 5 \succ _2 6 \succ _2 4 \succ _2 8 \succ _2 7. \end{aligned}$$

If ${{\mathcal {A}}}'=\{1,2,3,4\}$, then $\mathsf {worst}({{\mathcal {A}}}',v_1)=3$ and $\mathsf {worst}({{\mathcal {A}}}',v_2)=4$. $\square$

For the ease of case distinctions, we introduce another notion called *no later than* and observe a useful property regarding “no later than”.

#### Definition 6

(*No later than*) For two distinct alternatives *x* and *y*, we say that *x* is embedded *no later than y* if one of the following conditions holds.

(i) Alternatives *x* and *y* are both embedded during initialization.
(ii) They are both embedded in the same call to Refine().
(iii) When *y* is to be embedded, *x* is already embedded, i.e., $E(x)\ne \bot$.

We say that *y* is embedded *later than* *x* if (i) *x* is embedded no later than *y* while (ii) *y* is not embedded no later than *x*.

For instance, alternative 5 is embedded later than alternative 4 in Example 3 although they are embedded in the same iteration of the main loop.

The following lemma states that no alternative that is less preferred by both voters will be embedded too early, making sure that the inside-out approach in Refine() is correct.

#### Lemma 3

($*$) *Let* *x* *and* *y* *be two distinct alternatives with* $x \succ _1 y$ *and* $x \succ _2 y$. *Then, Algorithm 1 embeds x no later than y.*

Now, we continue with the correctness proof for the iteration stage. Let *D* be the alternatives that are embedded at the beginning of Refine or Fallback, and assume that *E* is one-dimensional Euclidean with respect to *D*.

In the remainder of the proof, we consider Refine() and Fallback() separately in Sects. 3.3.1 and 3.3.2.

#### Embedding alternatives in Refine($\succ _z,v_z,E$) with $z\in \{1,2\}$

Assume that Refine $(\succ _z,v_z,E)=\mathsf {true}$ as otherwise nothing needs to be proved. Let *C* be the set of alternatives that are to be embedded in the call and let $v_{z'}$ be the other voter with $z'\in \{1,2\}\setminus \{z\}$. By the procedure Refine($\succ _z,v_z,E$), the two embedded alternatives that Refine identifies are $c_{\mathsf {j}}$ and $c_{\mathsf {i}}$ in lines 16 and 18 such that $C=\{c_{{\mathsf {j}}},c_{{\mathsf {j}}+1},\dots ,c_{{\mathsf {i}}-1}\}$ and

1 $$\begin{aligned} c_{{\mathsf {j}}} \succ _z c_{{\mathsf {j}}+1} \succ _z \dots \succ _z c_{{\mathsf {i}}-1} \succ _z c_{{\mathsf {i}}} \succeq _z \mathsf {worst}(D,v_z). \end{aligned}$$

By assumption, the embedding *E* is one-dimensional Euclidean with respect to *D*.

**One-dimensional Euclideanness regarding voter** $v_z$. By Lemma 2, it follows that *E* is also a one-dimensional Euclidean embedding for voter $v_z$ regarding $D\cup C$. In particular, it holds that,

2 $$\begin{aligned} \forall k' \in \{1,2,\ldots , {\mathsf {i}}-1\}\!:\!|E(c_{k'})-E(v_z)| < |E(c_{k'+1})-E(v_z)|. \end{aligned}$$

**One-dimensional Euclideanness regarding voter** $v_{z'}$. It remains to show that *E* is also one-dimensional Euclidean for voter $v_{z'}$ regarding all alternatives from $D\cup C$.

To achieve this, we prove the following two lemmas which ensure that the newly embedded alternatives are one-dimensional Euclidean with respect to voter $v_{z'}$.

##### Lemma 4

($*$) *Assume that the input preference orders* $\succ _1$ *and* $\succ _2$ *are single-peaked.* *For each not-yet-embedded alternative* *x*, *i.e.,* $x\notin D$, *it holds that* $\mathsf {worst}(D,\succ _1)\succ _1 x$ *or* $\mathsf {worst}(D,\succ _2) \succ _2 x$.

The next lemma states that for each two not-yet-embedded alternatives, the single-peaked property imposes that they are ordered in the same way by both voters.

##### Lemma 5

($*$) *Assume that the input preference orders* $\succ _1$ *and* $\succ _2$ *are single-peaked. For each two not-yet embedded alternatives* *x* *and* *y*, *i.e.,* $x,y\notin D$ *with* $x\ne y$, *the following holds:*

*For each* $r\in \{1,2\}$ *it holds that* *if* $x\succ _r y \succ _r \mathsf {worst}(D,\succ _r)$, *then* $\mathsf {worst}(D,\succ _{s}) \succ _{s} x \succ _{s} y$, *where* $s \in \{1,2\}\setminus \{r\}$.

Now, if we apply Lemma 5 on the alternatives *C* in the preferences given in (1), we obtain that voter $v_{z'}$’s preferences must be $\mathsf {worst}(D,v_{z'})\succ _{z'} c_{{\mathsf {j}}} \succ _{z'} c_{{\mathsf {j}}+1} \succ _{z'} \cdots \succ _{z'} c_{{\mathsf {i}}-1}$. By the embedding of the alternatives from *C* (lines 22–23), for each alternative $a_{k}$ with ${\mathsf {j}}\le k \le {\mathsf {i}}-1$ it holds that

3 $$\begin{aligned} \text {if } z=1\text {, then } E(v_{z'})<E(v_z)<E(c_k)<E(c_{k+1}); \end{aligned}$$

4 $$\begin{aligned} \text {if } z=2\text {, then } E(c_{k+1})<E(c_k)<E(v_{z})<E(v_{z'}). \end{aligned}$$

In both cases, we obtain that

5 $$\begin{aligned} |E(c_k)-E(v_{z'})|<|E(c_{k+1})-E(v_{z'})|,~{\mathsf {j}}\le k \le {\mathsf {i}}-1. \end{aligned}$$

Thus, to show that *E* remains one-dimensional Euclidean for voter $v_{z'}$ regarding the alternatives from $D\cup C$, we only need to show that $|E(\mathsf {worst}(D,\succ _{z'}))-E(v_{z'})|<|E(c_{{\mathsf {j}}})-E(v_{z'})|$.

Now, if we can show that

6 $$\begin{aligned} \mathsf {worst}(D,\succ _{z'}) \succeq _z c_{{\mathsf {j}}-1}, \end{aligned}$$

then we can derive that

$$\begin{aligned} |E(\mathsf {worst}(D,\succ _{z'}))-E(v_{z'})|&\le |E(\mathsf {worst}(D,\succ _{z'})) -E(v_z)| +|E(v_z)-E(v_{z'})|\\&{\mathop {\le }\limits ^{(6)}} |E(c_{{\mathsf {j}}-1})-E(v_z)| +|E(v_z)-E(v_{z'})|\\&{\mathop {<}\limits ^{(2)}} |E(c_{{\mathsf {j}}})-E(v_z)| + |E(v_z)-E(v_{z'})|\\&{\mathop {=}\limits ^{(3),(4)}} |E(c_{{\mathsf {j}}})-E(v_{z'})|, \end{aligned}$$

which is what we needed to show.

Thus, the only remaining task is to show that (6) holds. We distinguish between two cases; let $\mathsf {best}(\succ _z)$ and $\mathsf {best}(\succ _{z'})$ denote the most-preferred alternative in $\succ _z$ and $\succ _{z'}$, respectively.

If $\mathsf {worst}(D,\succ _{z'}) \in \mathsf {inner}(\succ _1,\succ _2)$, then by the definition of inner alternatives, it follows that $\mathsf {worst}(D,\succ _{z'})\succeq _{z'} \mathsf {best}(\succ _z)$. By the definition of $\mathsf {worst}(D,\succ _{z'})$, this also implies $\mathsf {worst}(D,\succ _{z'})=\mathsf {best}(\succ _z)$, and thus $\mathsf {worst}(D,\succ _{z'}) \succeq _z c_{{\mathsf {j}}-1}$ as $\mathsf {best}(\succ _z)$ is the first alternative in $\succ _z$.

If $\mathsf {worst}(D,\succ _{z'}) \notin \mathsf {inner}(\succ _1,\succ _2)$, then $\mathsf {worst}(D,\succ _{z'})$ was embedded during an iteration stage in the main loop. Suppose, for the contradiction that $c_{{\mathsf {j}}-1}\succ _z \mathsf {worst}(D,\succ _{z'})$. By the definition of $c_{{\mathsf {j}}}$, it follows that

7 $$\begin{aligned} c_{{\mathsf {j}}} \succ _z c_{{\mathsf {j}}+1} \succ _z \cdots \succ _z c_{{\mathsf {i}}-1}\succ _z \mathsf {worst}(D,\succ _{z'}). \end{aligned}$$

Now, let us consider the iteration in the main loop where $\mathsf {worst}(D, \succ _{z'})$ was embedded. First of all, $\mathsf {worst}(D,\succ _{z'})$ cannot be embedded in Refine($\succ _{z'},v_{z'},E$) because by definition, there existed no other already-embedded alternative which is less preferred by voter $v_{z'}$. Second, neither can $\mathsf {worst}(D,\succ _{z'})$ be embedded in Refine($\succ _z,v_z,E$), since otherwise by (7) all alternatives from *C* must be embedded at least in the same call as $\mathsf {worst}(D,\succ _{z'})$, a contradiction. Finally, neither can $\mathsf {worst}(D,\succ _{z'})$ be embedded in Fallback($\succ _1, E$) because there existed at least one other alternative, namely $c_{{\mathsf {j}}}$, which is more preferred by $v_{z}$ to $\mathsf {worst}(D,\succ _{z'})$. This means that there exists no iteration where $\mathsf {worst}(D,\succ _{z'})$ can be embedded, a contradiction.

Summarizing, we have shown that $\mathsf {worst}(D,\succ _{z'}) \succeq _z c_{{\mathsf {j}}-1}$. This completes the proof for the case where alternatives are embedded in Refine($\succ _z,v_z,E$).

#### Embedding alternatives in Fallback($\succ _1,E$)

For brevity’s sake, define $a^*:=\mathsf {worst}(D,\succ _1)$ and $b^*:={\mathsf{worst}}(D,\succ _2)$. By our algorithm, it must hold that

8 $$\begin{aligned} D \succ _1 C \text { and } D\succ _2 C.\end{aligned}$$

We also infer that $C=\{a_{\mathsf {j}}\}$ where ${\mathsf {j}}=|D|+1$, and that

9 $$\begin{aligned} E(v_1)<E(a_{\mathsf {j}}). \end{aligned}$$

To show the one-dimensional Euclidean property, we only need to show that $|E(a^*)-E(v_1)| < |E(a_{\mathsf {j}})-E(v_1)|$ and $|E(b^*)-E(v_2)|< |E(a_{\mathsf {j}})-E(v_2)|$. By lines 28–29, it holds that $a^*=a_{{\mathsf {j}}-1}$. Thus, we infer that

10 $$\begin{aligned} |E(a^*)-E(v_1)| =|E(a_{\mathsf {j}})-E(v_1)|-1 <|E(a_{\mathsf {j}})-E(v_1)|. \end{aligned}$$

By the definition of $a^*$ and $b^*$, voter $v_1$ has preferences

11 $$\begin{aligned} b^* \succeq _1 a^*. \end{aligned}$$

Since *E* is one-dimensional Euclidean with respect to voter $v_2$ and the alternatives in *D*, this implies the following:

$$\begin{aligned} |E(b^*)-E(v_2)|&\le |E(b^*)-E(v_1)|+|E(v_1)-E(v_2)| \\&{\mathop {\le }\limits ^{(11)}} |E(a^*)-E(v_1)| + |E(v_1)-E(v_2)|\\&{\mathop {<}\limits ^{(10)}} |E(a_{\mathsf {j}})-E(v_1)|+|E(v_1)-E(v_2)|\\&{\mathop {=}\limits ^{(9)}} |E(a_{\mathsf {j}})-E(v_2)|. \end{aligned}$$

To conclude, we have shown that in each case the algorithm extends the embedding so that the resulting embedding is one-dimensional Euclidean for both voters and the alternatives already embedded as well as the newly embedded alternatives. Thus, our algorithm indeed computes a one-dimensional Euclidean embedding of two voters whose preferences are single-peaked.

## Single-peaked and single-crossing profiles with up to five alternatives are one-dimensional Euclidean

In this section, we state and prove our second main result concerning preference profiles with up to five alternatives.

### Theorem 3

*Each preference profile with up to five alternatives is one-dimensional Euclidean if and only if it is single-peaked and single-crossing.*

### Proof

From Observation 1, we know that a one-dimensional Euclidean profile is necessarily single-peaked and single-crossing. Thus, to show the theorem, it suffices to show that every single-peaked and single-crossing preference profile with up to five alternatives is also one-dimensional Euclidean. We achieve this by using a computer program via the CPLEX solver that exhaustively searches for all possible single-peaked and single-crossing profiles with up to five alternatives and provide a one-dimensional Euclidean embedding for each of them. Since the CPLEX solver accepts constraints on the absolute value of the difference between any two variables, our computer program is a simple one-to-one translation of the one-dimensional Euclidean constraints given in Definition 3. Hence, we do not need to compute any single-peaked or single-crossing order necessary for the non-trivial approaches as given in the literature (Doignon and Falmagne 1994; Knoblauch 2010; Elkind and Faliszewski 2014).

We did some optimization to significantly shrink our search space on all possible single-peaked and single-crossing preference profiles.

- First, we only consider profiles with at least two alternatives and at least two voters who have pairwise *distinct* preference orders as two voters with the same preference order can be embedded at the same position without losing the one-dimensional Euclidean property. Since the relevant profiles in consideration must be single-crossing, by Doignon and Falmagne (Doignon and Falmagne 1994, Lemma 1) and Bredereck et al. (Bredereck et al. 2013, Section 2.1), our program only searches for profiles with at most $$\left( {\begin{array}{c}m\\ 2\end{array}}\right) +1$$ distinct preference orders, where *m* is the number of alternatives, $3\le m\le 5$. The minimum number of voters we need to consider is three as by Theorem 1 all single-peaked and single-crossing preference profiles with two voters are one-dimensional Euclidean.
- Second, we assume that one of the preference orders in the sought profile is $1\succ 2 \succ \ldots \succ m$. We denote this order as the canonical preference order.
- Third, using the monotonicity of the single-peaked property, we consider adding a preference order (there are $m!-1$ many) to form a potential relevant single-peaked and single-crossing profile only if it is single-peaked with the canonical one. By Lackner and Lackner (Lackner and Lackner 2017, Theorem 12(i)), among all $m!-1$ preference orders other than the canonical one, there are $$\left( {\begin{array}{c}2m-2\\ m-1\end{array}}\right) -1$$ preference orders that each form with the canonical one a single-peaked profile. Note that for $m=5$, the number of potentially single-peaked profiles with $$n=\left( {\begin{array}{c}5\\ 2\end{array}}\right) +1=11$$ voters is reduced from $$\left( {\begin{array}{c}m!-1\\ n-1\end{array}}\right) =\left( {\begin{array}{c}119\\ 10\end{array}}\right) $$ to $$\left( {\begin{array}{c}\left( {\begin{array}{c}2m-2\\ m-1\end{array}}\right) -1\\ 10\end{array}}\right) =\left( {\begin{array}{c}69\\ 10\end{array}}\right) $$.

We summarize the number of single-peaked and single-crossing profiles with up to $m=5$ alternatives and up to $$n=\left( {\begin{array}{c}m\\ 2\end{array}}\right) +1$$ voters in Table 2. Note that we include profiles which have two voters although by Theorem 1 all single-peaked and single-crossing preference profile with two voters are one-dimensional Euclidean.

**Table 2:** For each number *m* of alternatives stated in the first column and for each number *n* of voters stated in the first row, $3 \le m \le 5$ and $$2 \le n \le \left( {\begin{array}{c}m\\ 2\end{array}}\right) +1$$, we summarize the number of single-peaked and single-crossing preference profiles we have produced that contain the canonical preference order $1\succ 2\succ \cdots \succ m$ and no two voters that have the same preference orders. For instance, when $m=3$ and $n=4$, the number of sought preference profiles is 2, as indicated in row two and column four

m	n									
	2	3	4	5	6	7	8	9	10	11
3	5	6	2	–	–	–	–	–	–	–
4	19	69	108	90	39	7	–	–	–	–
5	69	567	2124	4810	7185	7273	4964	2196	570	66

We implemented a program which, for each of these produced profiles, uses the IBM ILOG CPLEX optimization software package to check and find a one-dimensional Euclidean embedding. The verification is done by going through each voter’s preference order and checking the condition given in Definition 3. The source code and all generated profiles, together with their one-dimensional Euclidean embeddings and the distances used for the verification, are available online at https://owncloud.tuwien.ac.at/index.php/s/Pk8TZxva48LJt35 and can be verified using the software at https://owncloud.tuwien.ac.at/index.php/s/nysw13YkUajJpOn. $\square$

## Conclusion and outlook

We have shown that for preference profiles with at most five alternatives or at most two voters, being single-peaked and single-crossing suffices for being one-dimensional Euclidean.

Our research leads to some interesting followup questions. First of all, using our computer program from Sect. 4 we can produce all single-peaked and single-crossing preference profiles and all one-dimensional Euclidean preference profiles. A natural question is to count the number of structured (e.g., single-peaked, single-crossing, one-dimensional Euclidean) preference profiles and provide a closed formula in terms of the number *m* of alternatives and the number *n* of voters, in a similar spirit as recent work by Lackner and Lackner (2017) and Chen and Finnendahl (2018).

Second, both the single-peaked and the single-crossing property can be characterized by a few small forbidden subprofiles (Ballester and Haeringer 2011; Bredereck et al. 2013). However, this is not the case for the one-dimensional Euclidean property (Chen et al. 2017). Thus we ask: is it possible to characterize *small* one-dimensional Euclidean preference profiles via a few forbidden subprofiles? We note that one-dimensional Euclidean preferences can be detected in polynomial time, using linear programming (Doignon and Falmagne 1994; Knoblauch 2010). Chen (2016, Chapter 4.11) provided a generic construction and showed that there are at least *n*! single-peaked and single-crossing preference profiles with $n=m/2$ voters and *m* alternatives that are not one-dimensional Euclidean. For $m=6$, this number would be 6. However, through our computer program we found that for $m=6$ and $n=3$, out of 4179 single-peaked and single-crossing preference profiles, there are 48 which are *not* one-dimensional Euclidean. This gap in numbers merits further investigation.

Last but not least, for $d\ge 2$, *d*-dimensional Euclidean preference profiles are not necessarily single-peaked nor single-crossing (Bogomolnaia and Laslier 2007). In other words, the forbidden subprofiles that are used to characterize single-peaked or single-crossing preference profiles are not of use to characterize *d*-dimensional Euclidean profiles. Peters (2017) showed that finitely many small forbidden subprofiles are not enough to characterize the *d*-dimensional Euclidean property for any $d\ge 2$. This leads to the question of searching for compact, sufficient and necessary conditions for preference profiles to be *d*-dimensional Euclidean. Bogomolnaia and Laslier (2007) answered this question for profiles that may contain ties. Moreover, they showed that for each $d\ge 1$, there exists a non-*d*-dimensional Euclidean preference profiles with $d+2$ voters and $d+2$ alternatives. Bulteau and Chen (2020) used a computer program to verify that all preference profiles with up to seven alternatives and up to three voters are 2-dimensional Euclidean, and provided a preference profile with 3 voters and 28 alternatives which cannot be embedded into the two-dimensional space.

### Additional material for section 3.1

#### Proof of Lemma 2

*Lemma* 2. *Let* ${\mathsf {j}}$ *and* ${\mathsf {i}}$ *be as defined in a call to* Refine($\succ _z,v_z, E$) *with* $z\in \{1,2\}$. *If E satisfies *$|E(c_{{\mathsf {j}}-1})-E(v_z)| < |E(c_{{\mathsf {i}}})-E(v_z)|$, *then after the call to* Refine($\succ _z,v_z,E$) *it holds that* $|E(c_{{\mathsf {j}}-1})-E(v_z)|<|E(c_{\mathsf {j}})-E(v_z)|<\dots< |E(c_{{\mathsf {i}}-1})-E(v_z)|<|E(c_{{\mathsf {i}}})-E(v_z)|$.

##### Proof

By the definitions of ${\mathsf {j}}$ and ${\mathsf {i}}$, since *E* satisfies $|E(c_{{\mathsf {j}}-1})-E(v_z)| < |E(c_{{\mathsf {i}}})-E(v_z)|$ it holds that

12 $$\begin{aligned} \textsf {dist}({\mathsf {j}}-1)=|E(c_{{\mathsf {j}}-1})-E(v_z)| < |E(c_{\mathsf {i}})-E(v_z)| = \textsf {dist}({\mathsf {i}}), \end{aligned}$$

Note that $\textsf {dist}({\mathsf {j}}-1)$ and $\textsf {dist}({\mathsf {i}})$ are defined in lines 19–20 of Algorithm 1. From lines 22–23 of Algorithm 1, it is straightforward to verify that for each alternative $c_k$ with ${\mathsf {j}}-1\le k \le {\mathsf {i}}$,

13 $$\begin{aligned} |E(c_k)-E(v_z)|&= \textsf {dist}({{\mathsf {j}}-1}) + \frac{\textsf {dist}({{\mathsf {i}}})-\textsf {dist}({{\mathsf {j}}-1})}{{\mathsf {i}}-{\mathsf {j}}+1}\cdot (k-{\mathsf {j}}+1) \end{aligned}$$

Combining (13) with (12), we obtain the chain of inequalities in the lemma.$\square$

### Additional material for section 3.2

#### Continued discussion on the implementation for j and i

Now, we discuss in depth how we achieve the claimed running time of $O(m\cdot \mathsf {runtime}\text {-}\mathsf {mult}(m))$, using a few additional variables. An extended version of Algorithm 1 where we explicitly state how these variables are initialized and updated can be found in Algorithm 2. The differences are marked in red.

We use a counter ${\mathsf {s}}$, which counts the number of currently embedded alternatives, to test the condition in lines 10, 15, 27 of Algorithm 1 in constant time. Counter ${\mathsf {s}}$ is initialized with ${p-1}$; recall that the number of inner alternatives embedded during the initialization phase in Algorithm 1 is ${p-1}$.

We introduce two integer arrays $\mathsf {rk}_1$ and $\mathsf {rk}_2$ to store for each alternative *x* the position of *x* in the preference order of $\succ _1$ and $\succ _2$, respectively (see line 33 of Algorithm 2).

For each voter $v_z$, $z\in \{1,2\}$, we introduce two integer variables $u_z$ and $w_z$ which store the following information:

(i) $u_z$ stores the largest position (i.e., index) in $\succ _z$ among all alternatives that were embedded in the previous call to Refine($\succ _z,v_z,E$).
(ii) $w_z$ stores the largest position (i.e., index) in $\succ _z$ among all already embedded alternatives.

Both $u_1$ and $u_2$ are initialized to 1. $w_1$ is initialized such that $a_{w_1} = b_1$, while $w_2$ is initialized such that $b_{w_2}=a_1$ (see lines 31 and 40 of Algorithm 2). 
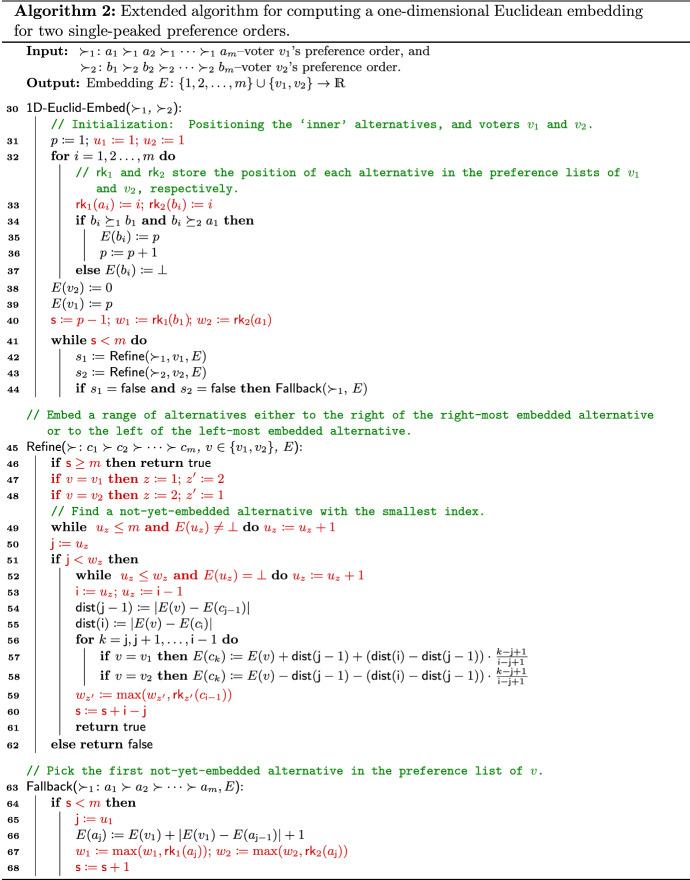


Assume that we are in a call to Refine($\succ _z,v_z,E$) with $\succ _z:c_1\succ _z c_2\succ _z \cdots \succ _z c_m$. To find ${\mathsf {j}}$, we go through the preference order of $v_z$, starting from $c_{u_z}$, and increment the value of $u_z$ until we find a not yet embedded alternative (see line 49 of Algorithm 2). Then, we set ${\mathsf {j}}:=u_z$ in line 50. We test the condition in line 17 in Algorithm 1 in constant time by checking whether $u_z < w_z$ since $c_{w_z}$ is the alternative with the largest index among all embedded alternatives. If the condition is met, then we find ${\mathsf {i}}$ by going through the preference order of $v_z$, starting from $c_{u_z}$ and incrementing the value of $u_z$ until we find a first already-embedded alternative (see line 52 of Algorithm 2). We set ${\mathsf {i}}:=u_z$ and $u_z:={\mathsf {i}}-1$ in line 53. We only need to update the value of $w_{z'}$ with $z'=3-z$ since the largest index among all embedded alternatives in the preference order of $v_{z'}$ may have changed (see line 59 of Algorithm 2). We update the counter ${\mathsf {s}}$ in line 60 of Algorithm 2.

We also need to adjust Fallback($\succ _1,E$). By the main loop, when we call Fallback(), both Refine($\succ _1,v_1,E$) and Refine($\succ _2,v_2,E$) must have returned $\mathsf {false}$. This also means that ${\mathsf {s}}< m$ and $u_1$ already points to a first not-yet-embedded alternative in the preference order of $v_1$. Hence, we only need to set ${\mathsf {j}}:=u_1$ in line 65 of Algorithm 2. We need to update the values of both $w_1$ and $w_2$ in line 67 of Algorithm 2, and update the counter ${\mathsf {s}}$ in line 68.

It is straightforward to see that the value of variable $u_z$ ($z\in \{1,2\}$) is never decreased and will always be increased by at least the number of alternatives to be embedded in each iteration. Since our algorithm terminates after at most $m-p+1$ calls of Refine, the search for ${\mathsf {j}}$ and ${\mathsf {i}}$ in all calls from the main loop combined needs *O*(*m*) time.

### Additional material for section 3.3

#### Proof of Lemma 3

*Lemma* 3. *Let x and* *y be two distinct alternatives with* $x \succ _1 y$ *and* $x \succ _2 y$.* Then, Algorithm 1 embeds x no later than y*.

##### Proof

If $y \in \mathsf {inner}(\succ _1,\succ _2)$, then by the transitivity of preference orders, it follows that $x\succ _1 y \succ _1 b_1$ and $x\succ _2 y \succ _2 a_1$, This immediately implies that $x\in \mathsf {inner}(\succ _1,\succ _2)$, meaning that *x* and *y* are both embedded during the initialization, and that *x* is embedded no later than *y*.

Now, let us assume that $y \notin \mathsf {inner}(\succ _1,\succ _2)$. Consider the call when *y* was embedded. There are two cases.

If *y* has been embedded in line 22 or line 23 in a call to Refine($\succ _z,v_z,E$), $z\in \{1,2\}$, then let ${\mathsf {j}}$ and ${\mathsf {i}}$ be the indices as defined in that call such that $c_{{\mathsf {j}}} \succeq _z y \succ _z c_{{\mathsf {i}}}$. If $E(x)\ne \bot$, i.e., *x* has already been embedded, then by the definition of “no later than”, *x* is embedded no later than *y*. If $E(x)=\bot$, since $c_{{\mathsf {j}}}$ was defined as the first alternative that is not yet embedded, it follows that $c_{{\mathsf {j}}} \succeq _z x$. Since $x \succ _z y$, it follows that $c_{{\mathsf {j}}} \succeq _z x \succ _z y \succ _z c_{{\mathsf {i}}}$, implying that *x* is embedded in the same call Refine$(\succ _z,v_z,E)$ as *y*. Thus, *x* is embedded no later than *y*.

If *y* has been embedded in Fallback($\succ _1,E$) in line 13, meaning that it is also the only alternative that is embedded during that iteration, then line 28 guarantees that $E(x)\ne \bot$, and thus, *x* is embedded no later than *y*.$\square$

#### Proof of Lemma 4

*Lemma* 4. *Assume that the input preference orders* $\succ _1$ *and* $\succ _2$ *are single-peaked*. *For each not-yet-embedded alternative* *x, i.e.,* $x\notin D$, it holds that $\mathsf {worst}(D,\succ _1)\succ _1 x$ *or* $\mathsf {worst}(D,\succ _2) \succ _2 x$.

##### Proof

Let $a^*=\mathsf {worst}(D,\succ _1)$ and $b^*=\mathsf {worst}(D,\succ _2)$. Towards a contradiction, suppose that $\succ _1$ and $\succ _2$ are single-peaked but *x* is an alternative with $x\notin D$ such that

14 $$\begin{aligned} x\succ _1 a^* \text { and } x\succ _2 b^*. \end{aligned}$$

This implies that

15 $$\begin{aligned} a_1 \ne a^* \text { and } b_1 \ne b^*, \end{aligned}$$

as $a_1 \succ _1 x$ and $b_1 \succ _2 x$; recall that $a_1$ (resp. $b_1$) is the alternative most preferred by voter $v_1$ (resp. $v_2$). Since *x* is not yet embedded while $a^*$ and $b^*$ are already embedded, applying Lemma 3 two times (letting $y=a^*$ and $y=b^*$, respectively), we know that

$$\begin{aligned} (a^*\succ _1 x \text { or } a^* \succ _2 x) \text { and } \,(b^*\succ _1 x \text { or } b^* \succ _2 x). \end{aligned}$$

Together, with the assumption given in (14), we obtain that

16 $$\begin{aligned}&v_1:b^*\succ _1 x \succ _1 a^* \text { and } v_2:a^* \succ _2 x \succ _2 b^*,\text { and thus, } \end{aligned}$$

17 $$\begin{aligned}&a^* \ne b^*. \end{aligned}$$

By the definitions of $a_1$ and $b_1$, we further infer that

18 $$\begin{aligned} v_1:a_1 \succeq _1 b^*\succ _1 x \succ _1 a^* \text { and } v_2:b_1 \succeq _2 a^* \succ _2 x \succ _2 b^*. \end{aligned}$$

We distinguish between two cases, in each case aiming to obtain $x\in \mathsf {inner}(\succ _1, \succ _2)$ which is a contradiction to $x\notin D$ as $\mathsf {inner}(\succ _1,\succ _2)\subseteq D$.

**Case 1:** If $a_1 = b^*$, then the preferences given in (18) are equivalent to

19 $$\begin{aligned} v_1:a_1 \succ _1 x \succ _1 a^* \text { and } v_2:b_1 \succeq _2 a^* \succ _2 x \succ _2 a_1. \end{aligned}$$

Furthermore, $b_1\ne a^*$ as otherwise $x\in \mathsf {inner}(\succ _1,\succ _2)$—a contradiction. Consequently, the preferences given in (19) imply that

20 $$\begin{aligned} v_1:a_1 \succ _1 x \succ _1 a^* \text { and } v_2:b_1 \succ _2 a^* \succ _2 x \succ _2 a_1. \end{aligned}$$

Since $\succ _1$ and $\succ _2$ are single-peaked, by Proposition 1 and by (20), we must have that $x\succ _1 b_1$. However, this implies that $x\in \mathsf {inner}(\succ _1, \succ _2)$ since $x\succ _2 a_1$—a contradiction.

**Case 2:** If $a_1 \ne b^*$, then the preferences given in (18) imply that

21 $$\begin{aligned} v_1:a_1 \succ _1 b^* \succ _1 x \succ _1 a^* \text { and } v_2:b_1 \succeq _2 a^* \succ _2 x \succ _2 b^*. \end{aligned}$$

Since $\succ _1$ and $\succ _2$ are single-peaked, by Proposition 1 and by (21), we must have that $x\succ _2 a_1$ and $x\succ _1 b_1$, implying that $x\in \mathsf {inner}(\succ _1, \succ _2)$—a contradiction.$\square$

#### Proof of Lemma 5

*Lemma* 5. *Assume that the input preference orders* $\succ _1$ *and* $\succ _2$ *are single-peaked. For each two not-yet embedded alternatives x* *and y , i.e.,* $x,y\notin D$ *with* $x\ne y$*, the following holds*:

*For each* $r\in \{1,2\}$ *it holds that*

*if* $x\succ _r y \succ _r \mathsf {worst}(D,\succ _r)$, *then* $\mathsf {worst}(D,\succ _{s}) \succ _{s} x \succ _{s} y$, *where* $s \in \{1,2\}\setminus \{r\}$.

##### Proof

Let $\succ _1$ and $\succ _2$ be single-peaked, and let *x*, *y* be as defined in the lemma. Consider an arbitrary index $r\in \{1,2\}$ such that $x \succ _r y \succ _r \mathsf {worst}(D,\succ _r)$ holds. Let $s\in \{1,2\}\setminus \{r\}$. Let $\mathsf {best}(\succ _z)$ be the most preferred alternative in the preference order $\succ _z$. Since $x,y\notin D$ and $\mathsf {best}(\succ _z)\in D$, we have that

22 $$\begin{aligned}&\mathsf {best}(\succ _r) \succ _r x \succ _r y \succ _r \mathsf {worst}(D, \succ _r). \end{aligned}$$

By Lemma 4, it follows that $\mathsf {worst}(D,\succ _s) \succ _s \{x,y\}$. Thus, it remains to show that $x\succ _s y$. Towards a contradiction, suppose that $y \succ _s x$. By the definition of $\mathsf {worst}(D, \succ _s)$, voter $v_s$ must have preferences

23 $$\begin{aligned} \{\mathsf {best}(\succ _r), \mathsf {worst}(D,\succ _r)\} \succeq _s \mathsf {worst}(D,\succ _s) \succ _s y \succ _s x. \end{aligned}$$

Together with (22), we have

$$\begin{aligned} \mathsf {best}(\succ _r) \succ _r x \succ _r y \succ _r \mathsf {worst}(D,\succ _r), \text { and } \{\mathsf {best}(\succ _r), \mathsf {worst}(D,\succ _r)\} \succ _s y \succ _s x, \end{aligned}$$

a contradiction to Proposition 1. $\square$

## Footnotes

1 https://www.ibm.com/analytics/cplex-optimizer.

2 The multiplication of two integers of *O*(*m*) bits can be done in $O(m\cdot \log (m) \cdot \log (\log (m)))$ time, for instance via the Schönhage–Strassen algorithm (Schönhage and Strassen 1971).
